# The desferrioxamine-prochlorperazine coma—clue to the role of dopamine-iron recycling in the synthesis of hydrogen peroxide in the brain

**DOI:** 10.3389/fnmol.2014.00074

**Published:** 2014-08-04

**Authors:** John Smythies, Lawrence Edelstein

**Affiliations:** ^1^Center for Brain and Cognition, Department of Psychology, University of California San DiegoLa Jolla, CA, USA; ^2^Medimark CorporationDel Mar, CA, USA

**Keywords:** dopamine, catecholamines, iron, superoxide dismutase, hydrogen peroxide, quinones, antioxidants, desferrioxamine

## Introduction

A major activity at glutamatergic synapses in the brain is the production of potentially toxic oxygen radicals, in particular superoxide, by postsynaptic enzymes such as PGH synthase and by mitochondria. These need antioxidant mechanisms, such as the local production of antioxidant molecules (such as glutathione and ascorbate) and antioxidant enzymes, for protection of the neuron and its component chemicals (Smythies, 1999a,b). There may be also a further mechanism for antioxidant protection of neurons, that has been little explored, and that is the O'Brien cycle. This paper will describe this cycle and present an hypothesis as to its *in vivo* function.

## The facts

For a baseline it will be convenient to give a brief account of the antioxidant defenses of the glutamate synapse. Here the main players in the cleft are ascorbate (the major hydrophilic extracellular antioxidant in brain), glutathione (the major lipophilic antioxidant), carnosine, and the antioxidant enzyme superoxide dismutase (SOD) (Smythies, 1999a). Reuptake of glutamate by the glutamate transporter is associated with a matching release of ascorbate into the cleft (Grünewald, 1993; Rebec and Pierce, 1994). Glutathione and SOD are actively secreted into the cleft by astrocytes (Stone et al., 1999). Presynaptic glutamate vesicles also contain the antioxidant peptide carnosine, which is released along with glutamate (Boldyrev et al., 1997). Lastly dopaminergic *boutons en passage* are closely border glutamatergic synapses: stimulation of D2 receptors results in the induction in the postsynaptic neuron of anti-oxidant enzymes important for redox cycling including SOD, gamma-glutamylcysteine synthase, glutathione synthase, glutathione peroxidase, glutathione S-transferase, and glutathione reductase (Tanaka et al., 2001; Smythies, 2002).

However, less data is available about the antioxidant protection of catecholamines in and around the synapse. In this regard it is important to note that the major mode of interneuronal communication by dopamine is by volume transmission—dopamine is released directly into the extracellular space modulated by the hydrophilic environment of the extracellular matrix (Rice and Cragg, 2008; Fuxe et al., 2013). Since dopamine is easily oxidized (Smythies, 1999b) this requires an efficient antioxidant system. There is evidence that this is partly provided by the hydrophilic molecule ascorbate:

— In the retina, Neal et al. (1999) showed that the release of ascorbate protects dopamine from oxidation.
— Using slow-scan voltammetry following the injection of ascorbate oxidase into the rat striatum, Rebec and Wang (2001) reported a rapid decline in both ascorbate and behavioral activation. Within 20 min, an ascorbate loss of 50–70% led to a near-total inhibition of all recorded behavior including open-field locomotion, approach of novel objects and social interaction with other rats.
— Using microdialysis, Morales et al. (2012) showed that, in the striatum, ascorbate release prevents dopamine oxidation. They also found that glutamate release inhibited dopamine uptake, while dopamine release inhibited glutamate release. However, this system is exceedingly complex. Hara et al. (2009) presented evidence that, under some circumstances involving cooperation with iron, ascorbate can act as a pro-oxidant and stimulate the generation of the highly neurotoxic hydroxyl radical.

Other than this, there is scant information of other antioxidant mechanisms in this system. However, a clue is offered from an obscure clinical source.

## An unexplained clinical syndrome: the desferrioxamine-prochlorperazine coma

Blake et al. (1985) reported that a combination of normal doses of the iron chelator desferrioxamine and the antiemetic D2-receptor blocker prochlorperazine, given to two patients with rheumatoid disease but otherwise normal iron metabolism, induced a deep coma that lasted for 2 days in one case and 3 days in the other. Their EEG depicted increased slow waves. Neither drug in isolation produces any such effect at any dose. The same effect was obtained in rats, with the coma lasting approximately 7 h in normal subjects, and some 36 h in iron-deficient rats. To date, the mechanism producing this phenomenon remains obscure.

In a previous communication, Smythies (2011) suggested that this coma might be caused by disruption of the O'Brien cycle in the brain. This cycle is generated by the interaction of superoxide, dopamine and iron (Zhao et al., 1998; Siraki et al., 2000). These workers demonstrated that, in isolated hepatocytes, cycling between ferrous and ferric iron linked to cycling between the catecholamine (in particular, dopamine) and its semiquinone—in the presence of superoxide—forms an effective dismuting system that transforms the superoxide into water and hydrogen peroxide. This cycle depends on the presence of free iron, superoxide and dopamine molecules at the same microanatomical locus (for background information see Smythies, 1999b). Therefore, the question can be asked: where in the brain could such propinquity exist?

Iron is a highly reactive molecule, and the only place within a neuron that free iron is known to exist is in endosomes, where, however, free dopamine is not to be found. With that said, there may be an alternate location worthy of consideration, that being the extracellular space between neurons. Here, as we have noted above, dopamine is present and functioning in its volume transmission role, as well as superoxide derived from NADPH-oxidase on the extracellular side of cell membranes (Oakley et al., 2009), from extracellular microglia (Zoccarato et al., 2005) and from mitochondria (Bao et al., 2009). NADPH-oxidase is widely distributed in the brain (Oury et al., 1999). Iron is also present but is ensconced its carrier molecule, transferrin. However, the affinity of catecholamines for iron is greater than that for transferrin, and Sandrini et al. (2010) have shown that catecholamines can “steal” ferric iron from transferrin by forming catecholamine-iron complexes. Therefore, it is quite possible that the O'Brien cycle could operate within the dopamine-containing extracellular space in the brain.

It is well established that dopamine oxidation products, such as dopamine quinone and dopaminochrome, that play a role in the O'Brien cycle, can be highly neurotoxic (Smythies, 1999b). Furthermore, dopamine activates MAO activity, and that, when in presence of ALDH2 deficiency, this activation can be toxic, particularly to mitochondria, due to the formation of H_2_O_2_ and toxic aldehydes (Kaludercic et al., 2014). However, our hypothesis is not concerned with the neurotoxic role of dopamine and its quinones (when they are not engaged in the cycle), but with their role in reducing toxic levels of superoxide and providing adequate levels of hydrogen peroxide (when they are active in the cycle). The O'Brien cycle merely recycles dopamine and its quinones with no effect on the levels of either. Therefore, considerations of dopamine and dopamine quinone toxicity *per se* lie outside the scope of this paper.

Another aspect of redox mechanisms at the dopamine synapse may be related to its co-transmission with glutamate. Most if not all dopaminergic neurons in the midbrain utilize glutamate as a co-transmitter (Descarries et al., 2008; Koos et al., 2011). Activation of dopaminergic neurons elicits small amplitude postsynaptic glutamatergic currents in all spiny pyramidal neurons in the nucleus accumbens, mediated by both AMPA and NMDA receptors. This is accompanied the by simultaneous release of dopamine. VGluT2 knockout has complex effects on risk-taking behaviors and anxiety (Koos et al., 2011). These authors list three possible functions mediated by this system: (1) various local postsynaptic effects, (2) glutamate release from these terminals may function as a heterosynaptic modulator of other inputs, and (3) the development and/or maintenance of dopaminergic synapses. In the case of the third possibility, one detail that may be implicated consists of the redox factors described above. An added benefit to dopaminergic transmission from co-transmission with glutamate may be the availability of the extensive antioxidant resources of the glutamate synapse listed earlier. In other words, if these neurons use both dopamine and glutamate as their transmitters, then the antioxidant mechanisms associated with the glutamate part may also be available to the dopamine part.

## Our hypothesis

Our hypothesis for the cause of the desferrioxamine-prochlorperazine coma is predicated on the fact that the brain has two powerful antioxidant-protective mechanisms in play that both involve dopamine. The first mechanism is the activation of a series of antioxidant enzymes by the dopamine D2-receptor, which is blocked by prochlorperazine. The second is the O'Brien cycle that would collapse if deprived of iron by desferrioxamine. The brain may be able to operate in the absence of either of these mechanisms, but not both. A series of experiments are indicated to test this hypothesis. These would include repeating the rat experiment by Blake et al. (1985) using a range of dopamine receptor blockers and iron chelation agents.

The implications of confirming that the O'Brien cycle is present and active in the brain are broad, and would extend well beyond a simple redox function or control of superoxide toxicity. The main product of the O'Brien cycle is hydrogen peroxide. This molecule is involved in a wide range of signaling pathways in the brain. For example:

— Modulation of both GABAergic and glutamatergic functions (Frantseva et al., 1998; Sah and Schwartz-Bloom, 1999).
— Blockade of catecholamine and glutamate uptake by synaptic vesicles (Wang and Floor, 1998).
— Inhibition of synaptic dopamine release (Chen et al., 2001).
— Inhibition of an adenosine-mediated synaptic transmission in hippocampal slices (Masino et al., 1999).
— Maintenance of essential cell populations in the brain (Dickinson et al., 2011).
— Modulation of membrane and cytoskeletal properties in astrocytes and increasing their intercellular connections (Zhu et al., 2005).

A general review of this topic is provided by Rice (2011). Conceivably, the action of dopamine in the O'Brien cycle might allow it to modulate the supply of hydrogen peroxide for each of these mechanisms. In and of itself, this would have far-reaching functional consequences in the context of central nervous system physiology and pathology.

### Conflict of interest statement

The authors declare that the research was conducted in the absence of any commercial or financial relationships that could be construed as a potential conflict of interest.
